# Supplementary material for: Cross-Cultural Adaptation and Psychometric Testing of the International Sedentary Assessment Tool for the Spanish Population
Source: Int J Environ Res Public Health. 2020 Jan 24;17(3):758. doi: 10.3390/ijerph17030758 (PMC7037491; doi:10.3390/ijerph17030758)
Supplement: Supplementary file 1 [file ijerph-17-00758-s001.pdf]

International Journal of Environmental Research and Public Health

MDPI

Supplementary Material 1-English Version

International Sedentary Assessment Tool (ISAT) – WEEKDAY

The following questions are about activities you did over the ***past week while sitting, reclining or lying down****. Do not count the time you spent in bed sleeping or napping.*

For questions 1-4 only count the time when this was your main activity. For example if you are watching television and surfing the Internet, count it as television time or computer time, but not as both. This does not apply to questions 5 or 6.

On a typical WEEKDAY in the past week, how much time did you spend sitting or lying down and…

SEDENTARY ITEM	TIME	
1. Watching TV or using a computer, tablet or smartphone. *(Count time watching videos, playing computer games, emailing or using the Internet. Do not include time spent on a computer at work or at school.)*	__ hours	_____ min
2. Watching television or videos. *(Count time spent watching television, DVDs and online videos)*	__ hours	_____ min
3. Using a computer. *(Count time spent on things such as computers, laptops, Xbox, PlayStation, iPod, iPad or other tablet, or a smartphone, YouTube, Facebook or other social networking tools, and the Internet).*	__ hours	_____ min
4. Sitting reading a book or magazine *(Only include reading during free time. Include reading done using electronic formats. Include time spent reading as part of your homework, but do not include time spent reading at work, during class time or while exercising).*	__ hours	_____ min
5. During the last 7 days, how much time did you usually spend sitting on a weekday? *(Include time spent at school or work, at home, while doing course work, and during leisure time. This may include time spent sitting at a desk, visiting friends, reading or sitting or lying down to watch television).*	__ hours	_____ min
6. Sitting or driving in a car, bus, or train.	__ hours	_____ min

International Sedentary Assessment Tool (ISAT) – WEEKEND

The following questions are about activities you did over the ***past week while sitting, reclining or lying down****. Do not count the time you spent in bed sleeping or napping.*

For questions 1-4 only count the time when this was your main activity. For example if you are watching television and surfing the Internet, count it as television time or computer time, but not as both. This does not apply to questions 5 or 6.

On a typical WEEKEND DAY in the past week, how much time did you spend sitting or lying down and…

**SEDENTARY ITEM**	**TIME**	
1. Watching TV or using a computer, tablet or smartphone. *(Count time watching videos, playing computer games, emailing or using the Internet. Do not include time spent on a computer at work or at school.)*	__ hours	_____ min
2. Watching television or videos. *(Count time spent watching television, DVDs and online videos)*	__ hours	_____ min
3. Using a computer. *(Count time spent on things such as computers, laptops, Xbox, PlayStation, iPod, iPad or other tablet, or a smartphone, YouTube, Facebook or other social networking tools, and the Internet).*	__ hours	_____ min
4. Sitting reading a book or magazine *(Only include reading during free time. Include reading done using electronic formats. Include time spent reading as part of your homework, but do not include time spent reading at work, during class time or while exercising).*	__ hours	_____ min
5. During the last 7 days, how much time did you usually spend sitting on a weekend day? *(Include time spent at school or work, at home, while doing course work, and during leisure time. This may include time spent sitting at a desk, visiting friends, reading or sitting or lying down to watch television).*	__ hours	_____ min
6. Sitting or driving in a car, bus, or train.	__ hours	_____ min
